# Real-time MRI guided percutaneous transthoracic left ventricular access and closure

**DOI:** 10.1186/1532-429X-13-S1-O61

**Published:** 2011-02-02

**Authors:** Israel M Barbash, Christina E Saikus, Kanishka Ratnayaka, Anthony Z Faranesh, Ozgur Kocaturk, Jamie A Bell, Vincent Wu, William H Schenke, Michael C Slack, Robert J Lederman

**Affiliations:** 1 National Institute of Health, Bethesda, MD, USA; 2 Children's National Heart Institute, Washington, DC, USA

## Introduction and objective

Percutaneous transthoracic left ventricular (LV) access and closure would be an enabling technology for a wide range of structural heart, electrophysiologic and proximal aorta procedures. We propose a real-time MRI guided approach to access and close the LV using novel active devices.

## Methods

Sixteen Yorkshire swine underwent percutaneous transthoracic LV access and followed up to three months (n=8).

All procedures were guided by real-time bSSFP MRI (Espree, Siemens) using two slices along the needle trajectory and a short-axis slice for cardiac monitoring.

**Figure 1:**
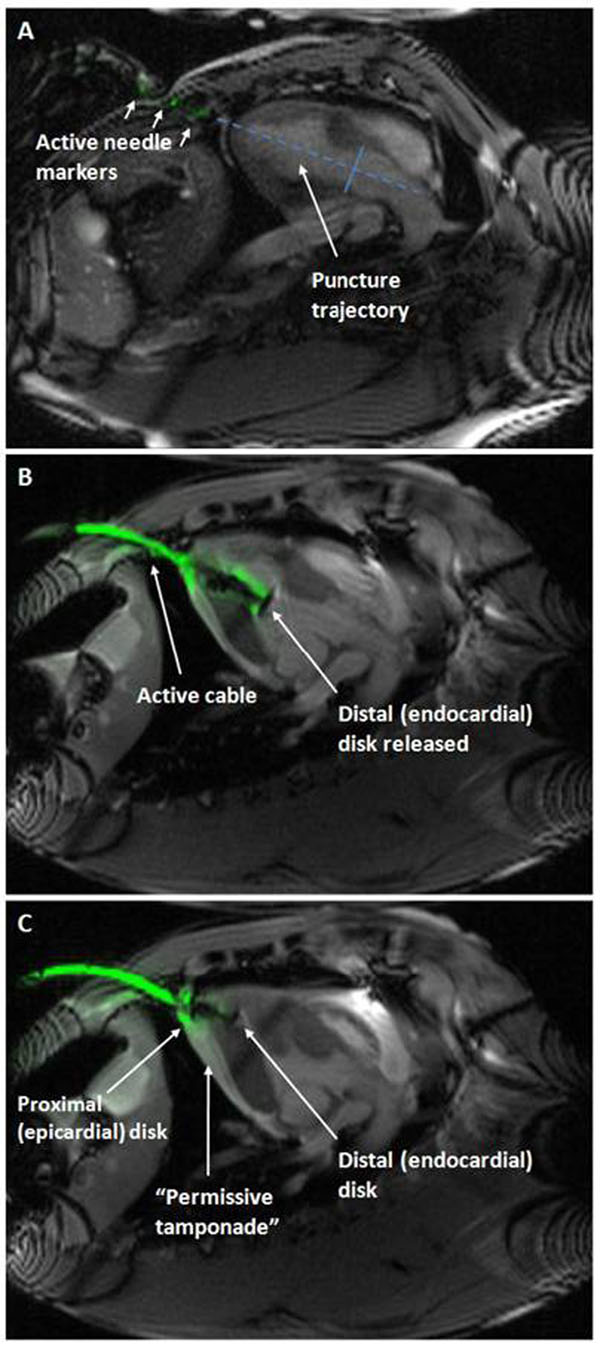


Transthoracic ventricular puncture was performed using a customized 18G active needle with integrated loop coil. Puncture trajectory was planned using real-time MRI to identify optimal LV puncture target and trajectory with respect to intraventricular and valvular structures (Figure 1A). After needle entry, an 18-French sheath was inserted and anticoagulation begun.

Ventricular puncture hole was closed using a modified Amplatzer muscular VSD occluder with a titanium screw to improve MR appearance. A custom active delivery cable incorporating a loopless antenna was used to enhance the visibility of the device deployment.

Occluder device was deployed by opening the distal disk at the endocardial surface (Figure 1B), separating pericardial layers by instillation of fluid into the pericardial space (“permissive pericardial tamponade”) in order to enable deployment of the proximal disk directly on top of the epicardial surface yet inside the pericardium (Figure 1C).

## Results

Real time MRI guidance enabled access to the LV without injuring intraventricular structures in 15/16 animals. In one animal the sheath injured the inter-ventricular septum.

Real-time MRI guided LV closure was achieved using the occluder device. Three types of failure modes of VSD occluder device mediated closure were observed during follow up. (1) Device was pulled out of the LV wall and resulted in fatality (n=1, day 0). (2) Pericardial entrapment by the proximal disc of the device resulted in continuous bleed and mortality (n=2, day 0, 12). (3) In one animal the puncture was apical and despite appropriate delivery, the device could not effectively occlude the puncture hole.

During follow-up (n=9), small pericardial effusion was aspirated on days 3-6 (196±127 ml). LV function was preserved and histology analysis indicated complete healing process around the device with no distant myocardial effects.

## Conclusions

Percutaneous transthoracic LV access and closure is a feasible approach using real time MRI and active devices. Transient “permissive pericardial tamponade” is important for effective deployment of the closure device.

